# Supplementary material for: Detection of Polymorphisms in FASN, DGAT1, and PPARGC1A Genes and Their Association with Milk Yield and Composition Traits in River Buffalo of Bangladesh
Source: Animals (Basel). 2024 Jun 30;14(13):1945. doi: 10.3390/ani14131945 (PMC11240816; doi:10.3390/ani14131945)
Supplement: Supplementary file 1 [file animals-14-01945-s001.zip › animals-3066771-supplementary.pdf]

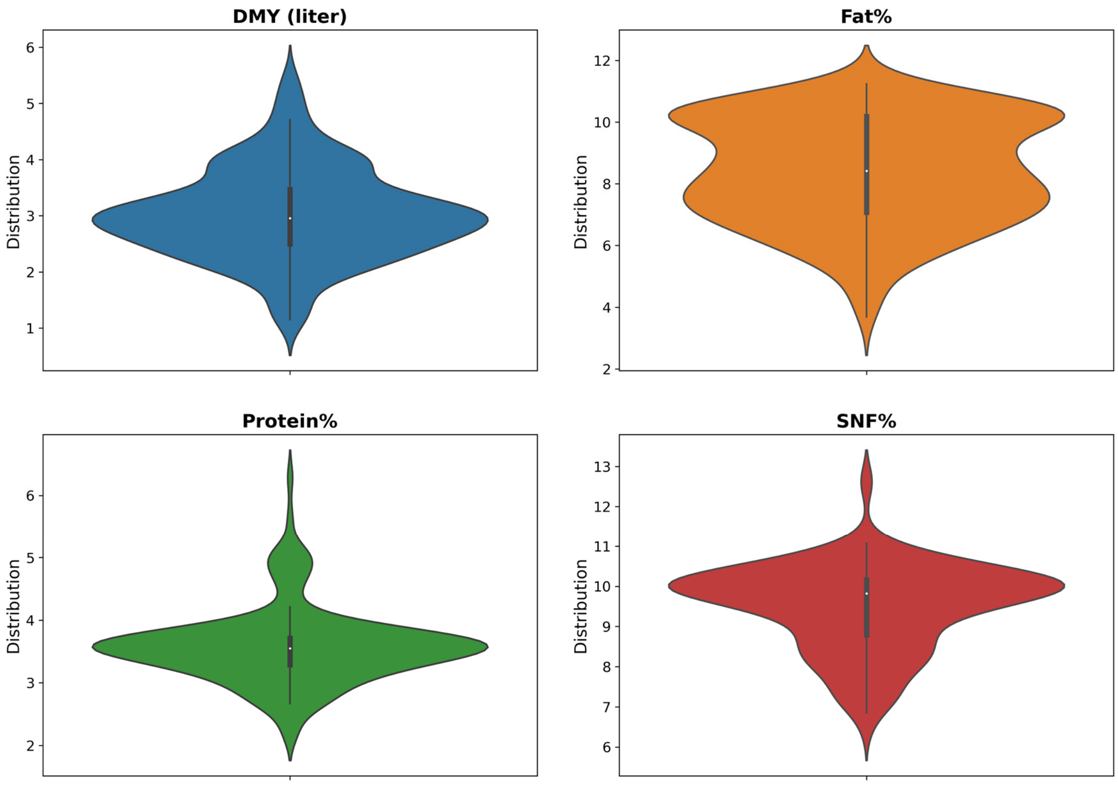

Figure S1: Violin plots of phenotypic data distribution for milk yield and composition traits in the river buffalo population of Bangladesh.
